# Gearbox Compound Fault Diagnosis in Edge-IoT Based on Legendre Multiwavelet Transform and Convolutional Neural Network

**DOI:** 10.3390/s23218669

**Published:** 2023-10-24

**Authors:** Xiaoyang Zheng, Lei Chen, Chengbo Yu, Zijian Lei, Zhixia Feng, Zhengyuan Wei

**Affiliations:** 1 School of Artificial Intelligence, Chongqing University of Technology, Chongqing 401135, China; 51212315120@stu.cqut.edu.cn (L.C.); rax_lie@stu.cqut.edu.cn (Z.L.); fengzhixia@stu.cqut.edu.cn (Z.F.); 2 School of Electronic and Automation, Chongqing University of Technology, Chongqing 400054, China; yuchengbo@cqut.edu.cn; 3 School of Science, Chongqing University of Technology, Chongqing 400054, China; weizy@stu.cqut.edu.cn

**Keywords:** compound fault diagnosis, edge-IoT, gearbox, Legendre multiwavelet transform, convolutional neural network

## Abstract

The application of edge computing combined with the Internet of Things (edge-IoT) has been rapidly developed. It is of great significance to develop a lightweight network for gearbox compound fault diagnosis in the edge-IoT context. The goal of this paper is to devise a novel and high-accuracy lightweight neural network based on Legendre multiwavelet transform and multi-channel convolutional neural network (LMWT-MCNN) to fast recognize various compound fault categories of gearbox. The contributions of this paper mainly lie in three aspects: The feature images are designed based on the LMWT frequency domain and they are easily implemented in the MCNN model to effectively avoid noise interference. The proposed lightweight model only consists of three convolutional layers and three pooling layers to further extract the most valuable fault features without any artificial feature extraction. In a fully connected layer, the specific fault type of rotating machinery is identified by the multi-label method. This paper provides a promising technique for rotating machinery fault diagnosis in real applications based on edge-IoT, which can largely reduce labor costs. Finally, the PHM 2009 gearbox and Paderborn University bearing compound fault datasets are used to verify the effectiveness and robustness of the proposed method. The experimental results demonstrate that the proposed lightweight network is able to reliably identify the compound fault categories with the highest accuracy under the strong noise environment compared with the existing methods.

## 1. Introduction

With the emergence of IoT, a large amount of innovative applications in fault diagnosis fields have been rapidly increasing [1]. For instance, Kumar et al. [2] proposed a fault diagnosis method based on IoT and semi-supervised learning for a panel-level solar photovoltaic array. Tran et al. [3] studied a novel fault recognition based on IoT and deep learning for induction motors. However, the existing centralized cloud computing models find it very difficult to cope with the massive number of IoT devices applied to the acquisition of fault data of rotating machinery and the long distance data transmission between devices and clouds. Consequently, it is very important to develop fast fault diagnosis methods of rotating machinery to avoid major safety accidents and economic losses in industrial production. Fortunately, the edge computing technique operated in the smaller number of IoT devices provides a promising direction addressing the deficiency of the centralized cloud computing [4]. For example, Wang et al. [5] proposed a lightweight convolutional neural network method for the intelligent fault diagnosis of bearing in the Industrial IoT context. Pan et al. [6] proposed a novel edge-IoT framework based on blockchain and smart contracts. Huang et al. [7] studied the development and application of multi-source sensing data fusion models and algorithms in mechanical equipment fault diagnosis and prediction based on IoT with artificial intelligence and big data processing technology.

For rotating machinery, gearbox is the most important power transmission component in mechanical equipment, which mainly consists of gears, shafts and bearings. Its health status directly affects whether the mechanical equipment can work normally. Due to different types of faults coupled together, non-stationarity and a large amount of noise, it is very difficult to effectively extract the most valuable fault characteristics from the raw data by using the existing methods [8]. If the specific fault category can be accurately recognized and predicted in the edge-IoT context, then the huge losses caused by the fault should be effectively avoided [9]. Thus, it is significantly meaningful to develop a high accuracy fault diagnosis method for the gearbox compound faults under a strong noise environment.

It is known that feature extraction and identification of the fault patterns are the two main steps to accomplish the fault diagnosis of rotating machinery [10]. Usually, the traditional feature extraction methods mainly consist of statistical feature extraction [11], signal analysis techniques such as Fourier transform [12], wavelet transform [13], empirical modal decomposition [14], and more. Then, the typical pattern recognition methods include support vector machines [15], extreme learning machines [16], artificial neural networks [17] and other improved approaches [18]. For example, Wang et al. [18] completed the diagnosis of the gearbox compound faults by using a double-extreme learning machine to implement the process of clustering and classification, respectively.

Although the traditional fault diagnosis methods have achieved some satisfactory results, there still exist many shortcomings. In summary, firstly, the traditional methods largely rely on expert knowledge and prior knowledge to obtain high quality features. Secondly, the traditional approaches typically exhibit poor generalization ability and lack high diagnostic accuracy, as they are easily influenced by environmental factors such as the strong noise interference [19].

In recent years, some new intelligent diagnosis methods based on deep learning have been widely used in the gearbox fault diagnosis fields [19], which have a strong self-learning ability and can obtain distinguishable fault features from the raw data after multiple iterations of learning [20]. For example, Autoencoder [21], convolutional neural networks [22], residual neural networks [23], recurrent neural networks [24], long short-term memory neural networks [25], and more, are implemented to identify the fault categories of rotating machinery. It is noted that the convolutional neural network and graph attention network have been widely applied in various research fields characterized by high computational data requirements due to their powerful modeling representation capability [26,27]. In addition, there is also the use of transfer learning to investigate deep network models, which can adaptively recognize various faults [28].

However, the diagnostic methods based on deep learning are largely dependent on hardware and high training cost, and the models often do not have strong generalization capability or anti-noise ability. It is noted that some researchers combined signal processing methods with deep learning to develop more effective fault diagnosis methods, which are more robust and have less learning cost [29]. For example, Bai et al. [30] used Fourier transform to process the sensor signal into an image and then applied a MCNN to mine fault characteristics. Chen et al. [31] utilized wavelet transform to decompose the raw data and then identified the internal features through a MCNN and a softmax classifier. Hong et al. [13] decoupled the compound faults signals by balanced multiwavelets and maximum correlated kurtosis deconvolution and then extracted the fault frequencies by spectrum analysis. But, it is greatly difficult to obtain high diagnosis accuracy in the situation of accurately locating the specific fault type from the compound faults, especially good robustness against noise under the strong noise conditions by using the existing methods.

To summarize, the difficulties of the existing methods in gearbox compound fault diagnosis mainly lie in three aspects. First, the complexity of the compound faults with highly non-stationary and a large amount of noise usually leads to attain low diagnosis accuracy for locating the specific fault type. Second, the traditional methods largely depend on artificial feature extraction and more complicated algorithms to select the most valuable features. Third, the deep learning-based methods need more complex model architectures and extensive training to finish the compound fault diagnosis. Especially, the ability of the extraction feature based on the deep models is significantly affected by the strong noise.

In view of the problems mentioned above, a novel and high accuracy fault diagnosis method, LMWT-MCNN, is proposed in this work for the gearbox compound faults. The proposed method decomposes the raw data into a few low and high frequency components using LMWT. Then, the feature images are designed based on these frequency components. Finally, the powerful feature learning ability of the MCNN model is implemented to further extract the more salient and valuable fault features from the feature images without artificial feature selection.

It is noted that LMWT has more base functions and many excellent properties to match the complex fault characteristics of gearbox. Therefore, the feature images obtained by LMWT can effectively represent the discriminative fault characteristics of gearbox, and there is no redundancy and leakage due to its orthogonality. Furthermore, the amplitude of the noise in the feature images is usually smaller than that of the fault frequency components [32], thus the process of the max pooling layers in the MCNN model can effectively remove the noise frequency components, which demonstrates the strong anti-noise ability of the proposed method. In addition, the proposed method uses multiple labels to effectively identify the specific fault type of the gearbox compound faults [33].

Finally, the effectiveness and robustness of the proposed method are verified by the PHM 2009 dataset from the 2009 Prognostics and Health Management Competition (https://phmsociety.org/data-analysis-competition/, accessed on 21 October 2023) [34] and the Paderborn University bearing compound fault datasets (https://mb.uni-paderborn.de/en/kat/main-research/datacenter/bearing-datacenter/data-sets-and-download/, accessed on 21 October 2023) [35]. The two datasets are conducted by some fault diagnosis methods but it is difficult to achieve high diagnostic accuracy [14,25,33,36,37,38,39,40,41,42,43].

However, the experimental results obtained in this paper demonstrate that the proposed method has the great merits of the highest diagnosis accuracy and more robustness than other existing methods. In summary, the main contributions and advantages of this paper are described as follows.

(1) This paper constructs two feature images based on LMWT frequency domain by using a sample data, which can effectively match the complex fault characteristics of rotating machinery.
(2) This paper proposes an end-to-end compound fault diagnosis model based on edge-IoT. The proposed model not only avoids the complex artificial feature extraction, but also is a lightweight network only consisting of three convolutional layers and corresponding three pooling layers.
(3) This paper provides an effective model for extracting multiple fault features in the strong noise environment and it is very suitable for the compound fault diagnosis in real applications.
(4) This work conducts some comparative experiments on two datasets of rotating machinery, which verifies the effectiveness and robustness of the developed method. The corresponding recognition results indicate that the proposed model achieves the highest diagnosis accuracy and shows powerful anti-noise ability.

The remainder of this paper is organized as follows: Section 2 introduces edge-IoT, LMWT, and the CNN model. The decomposition and reconstruction of a sample of the gearbox fault case 2 are elaborately described for understanding how to decompose the raw data into different frequency components by LMWT. In Section 3, the two feature images of a sample are constructed based on the frequency components in detail. Then, the flowchart of the hybrid fault diagnosis method of LMWT and MCNN models based on edge-IoT is elaborately described. In Section 4, the proposed method is implemented to identify different fault categories of rotating machinery, and the diagnosis results are utilized to compare with the existing methods. Finally, Section 5 gives some conclusions about this research and prospects for future work.

## 2. Research Methodology

In this section, the framework of edge-IoT is first described in detail. In the second step, the concept and properties of LMW bases are introduced, and the decomposition and reconstruction of a sample are specifically described in this context. In the third step, the structure of CNN model is elaborately described. Finally, the multi-label method for gearbox compound fault diagnosis is briefly introduced.

### 2.1. The Data Acquisition and Fault Diagnosis System Based on Edge-IoT

**Figure 1:**
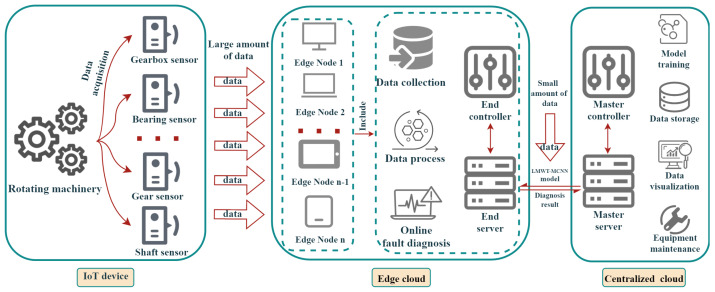
Rotating machinery compound fault data acquisition and fault diagnosis system based on edge-IoT.

It is known that the mechanical equipment intelligent fault diagnosis mainly consists of three processing procedures: signal acquisition, feature extraction and classification diagnosis. The data acquisition stage has a significant impact on the industrial application of mechanical fault diagnosis. Traditional fault diagnosis systems are mostly based on the centralized cloud computing structure [1]. However, if the data volume of the terminal is large, the centralized transmission of data based on the centralized cloud has high requirements for the bandwidth of the transmission network, which will consume huge bandwidth and computing resources [4]. Wu et al. [44] proposed the edge-cloud architecture for IoT devices with the function of mechanical equipment intelligent diagnosis, which can effectively cope with the difficulty of the large volume data of the terminal and arrive the requirement for online fault diagnosis. Consequently, this paper adopts a data acquisition and intelligent fault diagnosis systems based on edge-IoT for gearbox compound faults, which is demonstrated in Figure 1 as follows.

As shown in Figure 1, different sensor groups are used to collect the equipment fault data of rotating components such as gearbox, bearing, gear, wind turbines and other mechanical equipment in different environments. Then, the end controller receives the large amount of data attained by the device, and the end server receives the proposed model from the master server. The collected large amount of data are transported to the edge calculation node, which has the proposed lightweight model for online fault diagnosis. The proposed end-to-end lightweight network is effectively utilized to attain the highest accuracy fault diagnosis results on the edge computing nodes. Finally, the obtained diagnosis results with a small amount of data are transported into the centralized cloud platform to be analyzed and visualized by the master controller.

To summarize, the edge computing lightweight model based on IoT can process sensor data directly at the edge of the network, which not only meets the expansion needs of the computing power of terminal devices, but also solves the issue of long delay in accessing cloud data centers. Compared with the centralized cloud computing, the proposed fault diagnosis method based on edge-IoT makes data analysis, communication, control, and storage closer to the sensing point, with low delay, less energy consumption, and high accuracy performance.

### 2.2. Legendre Multiwavelet Bases

Legendre polynomials of degree *k* denoted by $L_{k}\left(x\right)$ are described as

(1) $$\begin{array}{c} L_{0}\left(x\right)=1,\;\;\;\;L_{1}\left(x\right)=x,\\ L_{k+2}\left(x\right)=\frac{2k+3}{k+2}xL_{k+1}\left(x\right)-\frac{k+1}{k+2}L_{k}\left(x\right), \end{array}$$

where k=0,1,⋯,p−1, and *p* is the number of the adopted LMW bases. According to the literature [45], the Legendre scale basis functions $\phi_{k}\left(x\right)$ is represented by

(2) $$\phi_{k}\left(x\right)=\left\{\begin{array}{cc} \sqrt{2k+1}L_{k}\left(2x-1\right), & x\in [0,1],\\ 0, & x\notin [0,1]. \end{array}\right.$$

Furthermore, a subspace $V_{p,n}$ of piecewise polynomials is defined as

(3) $$V_{p,n}=\left\{f:f{|}_{I_{nl}}\;\mathrm{is}\;a\;\mathrm{polynomial}\;\mathrm{of}\;\mathrm{degreestrictly}\;\mathrm{less}\;\mathrm{than}\;p;\;f\;\mathrm{vanishes}\;\mathrm{elsewhere}\right\},$$

which constitutes a linear space, where n=0,1,⋯ is the resolution level, and $l=0,1,\cdots ,2^{n}-1$ is the translation parameter, and the corresponding interval $I_{nl}$ is represented by $I_{nl}=\left[2^{-n}l,2^{-n}\left(l+1\right)\right)$. It is obvious to the whole set ${\left\{\phi_{k}\right\}}_{k=0}^{p-1}$ forms an orthonormal basis for the subspace $V_{p,0}$. Then, the subspace $V_{p,n}$ is also spanned using $\phi_{k}$ by dilation and translation,

(4) $$V_{p,n}=\mathrm{span}\left\{\phi_{k,nl}\left(x\right)=2^{n/2}\phi_{k,n}\left(2^{n}x-l\right)\right\},$$

which forms an orthonormal basis in the subspace $V_{p,n}$. If the vibration signals with various faults of rotating machinery are analyzed only in the subspace $V_{p,n}$, the low frequency components are essentially obtained at the resolution level *n*. Whereas a lot of characteristics of rotating machinery are salient to the high frequency components, the orthogonal complement of $V_{p,n}$ in $V_{p,n+1}$, i.e., the multiwavelet subspace $W_{p,n}$ needs to be described as

(5) $$V_{p,n}⊕W_{p,n}=V_{p,n+1},\;V_{p,n}⊥W_{p,n}.$$

It is known that Alpert [45] has constructed this multiwavelet subspace, which is implemented to effectively compute the integral and differential operators. The corresponding results can be explained by the two scales relation of the form

(6) $$\phi_{k}\left(x\right)=\sqrt{2}\sum_{k^{\prime }=0}^{p-1}\left(h_{kk^{\prime }}^{\left(0\right)}\phi_{k^{\prime }}\left(2x\right)+h_{kk^{\prime }}^{\left(1\right)}\phi_{k^{\prime }}\left(2x-1\right)\right),$$

(7) $$\psi_{k}\left(x\right)=\sqrt{2}\sum_{k^{\prime }=0}^{p-1}\left(g_{kk^{\prime }}^{\left(0\right)}\phi_{k^{\prime }}\left(2x\right)+g_{kk^{\prime }}^{\left(1\right)}\phi_{k^{\prime }}\left(2x-1\right)\right),$$

where $\psi_{k}$ is the multiwavelet basis. In this work, the above coefficient matrices $H_{0}={\left(h_{ij}^{\left(0\right)}\right)}_{p\times p}$, $H_{1}={\left(h_{ij}^{\left(1\right)}\right)}_{p\times p}$, $G_{0}={\left(g_{ij}^{\left(0\right)}\right)}_{p\times p}$, and $G_{1}={\left(g_{ij}^{\left(1\right)}\right)}_{p\times p}$ are implemented to learn the fault characteristics by convolution of the rotating machinery fault data to facilitate thoroughly extraction of comprehensive features.

**Figure 2:**
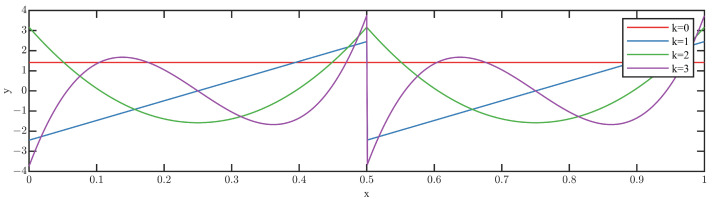
Legendre scale bases with n=1 and p=4.

**Figure 3:**
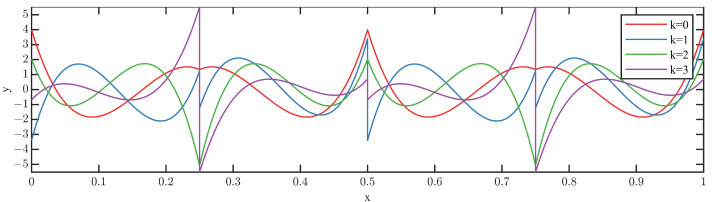
Legendre wavelet bases with n=1 and p=4.

In addition, in order to intuitively understand Legendre scale bases and wavelet basis functions, let the finest resolution level n=1 and order p=4, respectively, and plot these bases which are described in Figure 2 and Figure 3.

From Figure 2 and Figure 3, the rich properties, such as compact support, vanishing moments, orthogonality, various regularities are clearly shown, and LMWT provides a powerful tool for comprehensively extracting the fault characteristics of the rotating machinery data through a few Legendre scale and wavelet bases. Various regularities should be more appropriate to adaptively identify the complex fault characteristics instead of the traditional fault diagnosis methods that rely on engineering experience.

### 2.3. The Decomposition and Reconstruction of LMWT

LMWT can be considered as a mathematical tool that converts a signal into a series of scale and wavelet coefficients, respectively. According to the multiresolution analysis theory and the basis knowledge of LMW explained in the above subsection, the decomposition procedure j+1→j resolution level is based on

(8) $$s_{k,jm}=\sum_{k^{\prime }=0}^{p-1}\left(h_{kk^{\prime }}^{\left(0\right)}s_{k^{\prime },\left(j+1\right),2m}+h_{kk^{\prime }}^{\left(1\right)}s_{k^{\prime },\left(j+1\right),\left(2m+1\right)}\right),$$

(9) $$d_{k,jm}=\sum_{lk^{\prime }-0}^{p-1}\left(g_{kk^{\prime }}^{\left(0\right)}s_{k^{\prime },\left(j+1\right),2m}+g_{kk^{\prime }}^{\left(1\right)}s_{k^{\prime },\left(j+1\right),\left(2m+1\right)}\right),$$

where $s_{k,jm}$ and $d_{k,jm}$ are the low frequency and high frequency components at the resolution level *j*, i.e., the approximation coefficients and detail coefficients, respectively. The integer *m* is the number of the data obtained by the resolution level j+1 and $m=0,1,\cdots ,2^{j}$. Therefore, the signals are decomposed into a hierarchical structure of details and approximations at the finest resolution level *n* as follows.

(10) $$f:=\sum d_{k,jm}+s_{k,0m}.$$

Correspondingly, the reconstruction j+1→j resolution level is described as

(11) $$s_{k,(j+1),2m}=\sum_{k^{\prime }=0}^{p-1}\left(h_{kk^{\prime }}^{\left(0\right)}s_{k^{\prime },jm}+g_{kk^{\prime }}^{\left(0\right)}d_{k^{\prime },jm}\right),$$

(12) $$s_{k,(j+1),(2m+1)}=\sum_{k^{\prime }=0}^{p-1}\left(h_{kk^{\prime }}^{\left(1\right)}s_{k^{\prime },jm}+g_{kk^{\prime }}^{\left(1\right)}d_{k^{\prime },jm}\right).$$

**Figure 4:**
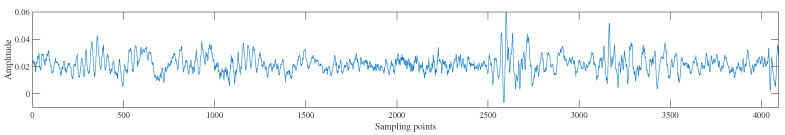
Raw data of gearbox with 4096 points for case 2.

Furthermore, a specific sample of the PHM 2009 dataset for case 2 is utilized to demonstrate the effectiveness and stability of LMWT. Then, the raw gearbox fault data of the sample with 4096 points for case 2 are described in Figure 4.

Specifically, the convolution procedure of LMWT with the order of wavelet bases p=2 is described by the above sample as follows.

**Figure 5:**
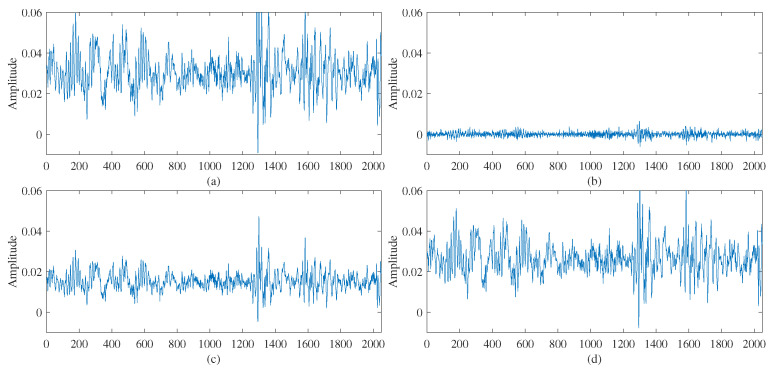
Low frequency (**a**,**c**) and high frequency (**b**,**d**) components for case 2 by LMW decomposition.

Step 1: The choice of finest resolution data is adopted as the raw data.
Step 2: According to the decomposition Equations (8) and (9), the raw data is doubled due to using two wavelet. Then, the doubled raw data is segmented into two parts corresponding to 2m and 2m+1, which are easily processed by the four filters.
Step 3: The processed data produce the correspondingly low frequency and high frequency components according to the decomposition Equations (8) and (9) at resolution level 1 by two Legendre scale bases and two Legendre wavelet bases.
Step 4: The detailed frequency components are elaborately demonstrated in Figure 5 as follows.

**Figure 6:**
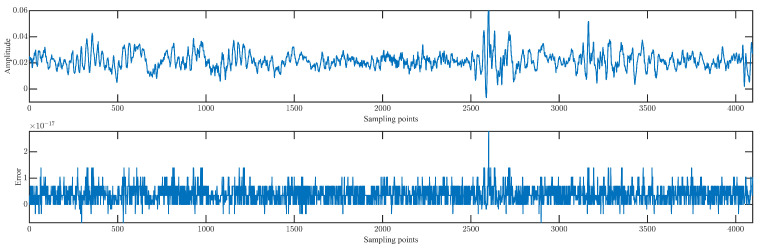
Reconstruction of the raw gearbox fault data and corresponding reconstruction error by LMWT.

As illustrated in Figure 5, the resolution level 1 by LMW decomposition (LMWD) generates a total of four frequency components without losing any frequency information because of orthogonality. Then, according to the reconstruction Formulas (11) and (12), the gearbox fault data for case 2 can be reconstructed with high accuracy and no Gibbs phenomena, and it is described in Figure 6.

As shown in Figure 6, the order of the magnitude of the reconstruction error is $10^{-17}$, which demonstrates the effectiveness and stability of this transformation.

### 2.4. A Brief Introduction to CNN

As one of the most important deep learning structure models, CNN model has been widely applied with great success to various fault recognition fields [46]. In this subsection, the structure of CNN model is first explained in detail. Then, the loss function is elaborately introduced.

The main structure of CNN model is a multi-layer network, which consists of one input layer, alternative convolutional layers and pooling layers, fully connected layers, and one output layer. The convolutional layers applied a number of convolutional kernels to serve as the local filters to slide over the whole input neurons at the previous layer for generating various feature maps. The convolutional operation between the input neurons and the learnable convolutional kernels can be described by

(13) $$x_{j}^{l}=\sigma \left(\sum_{i}x_{i}^{l-1}*k_{ij}^{l}+b_{j}^{l}\right),$$

where $x_{j}^{l}$ is $j^{th}$ feature map at the lth layer, $x_{i}^{l-1}$ denotes the ith input feature map at ${\left(l-1\right)}^{th}$ layer, $k_{ij}^{l}$ denotes the convolutional kernel which connected $i^{th}$ input feature map with $j^{th}$ feature map, $b_{j}^{l}$ denotes the bias, and ∗ denotes the convolutional operation. σ(·) is an activation function, such as the sigmoid function, hyperbolic tangent function and rectified linear units (ReLU). In contrast with the other activation functions, ReLU applies unilateral inhibition method to alleviate the risk of vanishing gradient problems and accelerate the convergence, which has been widely used in CNN model. The ReLU function is described as

(14) $$\mathrm{ReLU}\left(x\right)=\left\{\begin{array}{cc} x, & \;\mathrm{if}\;x>0,\\ 0, & \;\mathrm{if}\;x\leq 0. \end{array}\right.$$

Pooling layers are used to decrease the number of the neurons in the network and achieve low resolution of feature maps, which usually follow the convolution layers adjacently. In CNN model, max-pooling, average-pooling, and stochastic-pooling are the common operations in pooling layers. After multi-stage convolutional layers and pooling layers, a fully connected layer is added to integrate the discriminative local information of the category; in the full connection layer, dropout technology is often used as a regularization method to restrain overfitting.

Furthermore, extracted features of the convolutional layers are flattened and then inputted into the fully connected layers, which work in a similar manner as the traditional back-propagating neural network.

Finally, the output layer uses the classifier for data classification. In the classifier, the softmax function is adopted as the classifier to classify the normal and fault data. To be specific, the estimated probability denoted by $q_{c}\left(x\right)$ can be calculated as follows.

(15) $$q_{c}\left(x\right)=\mathrm{Prob}\left(y^{c}\right)=\frac{e^{y^{c}}}{\sum_{c^{\prime }=1}^{C}e^{y^{c^{\prime }}}},$$

where the observation *x* belongs to $c^{th}$ class, $y^{c}$ is the $c^{th}$ fault class in the full connected layers, and *C* is the number of the fault classes. Since the cross-entropy loss of CNN model can accelerate the updating speed of weights and convergence speed of the whole model in comparison with the squared error loss in common classification tasks, in this paper, the cross-entropy loss function is applied to diagnose the various fault categories of rotating machinery and is described as

(16) $$L\left(p\left(x\right),q\left(x\right)\right)=-\sum_{c=1}^{C}p_{c}\left(x\right)\log\left(q_{c}\left(x\right)\right),$$

which is implemented to measure the distance between output probability of the network and real target, i.e., the real probability $p_{c}\left(x\right)$.

In contrast with the traditional fully connected neural network, the CNN model is only sensitive to the local receptive field by employing sparse connections to a small scope of neurons, and applies a weight sharing strategy to decrease the number of parameters. Therefore, the CNN model can significantly decrease the computational burden of the whole network and make the network easier to train.

### 2.5. Multi-Label Approach for Compound Fault Diagnosis

The compound fault vibration signal possesses typical nonlinear and nonstationary properties, and the coupled fault characteristics are immersed in the strong noise. Thus, it is very difficult to effectively extract the coupled characteristics from the raw vibration signal.

The proposed model in this paper locates the compound faults by the multi-label method. To be specific, the label vector of each health condition is represented by multi-hot labels with 1 at multiple indices rather than single hot label. That is, the occurrence of the corresponding fault type is recorded as 1. Subsequently, a softmax layer serves as the output layer in the proposed architecture, where the output represents the probability of each type of fault occurrence. If the position with the highest probability in the network output is the same as the position of 1 in the multi-label, the diagnosis result is regarded as the situation of correction.

Finally, a cross-entropy loss function is implemented to calculate the loss value by the comparison between the output and the multi-label value for updating network parameters. This labelling method can locate the specific fault type of rotating machinery compound faults, where the specific fault type is effectively distinguished through the trained network model.

## 3. The Proposed Method

In this section, the feature images obtained from the gearbox compound fault data by LMWD are first devised. Then, a two-channel CNN model based on the feature images is elaborately described. Finally, the proposed method based on edge-IoT is clearly explained. Correspondingly, their flowcharts are specifically explained in Section 3.1, Section 3.2 and Section 3.3, respectively.

### 3.1. Constructing Feature Images by LMWD Frequency Domain

In this subsection, the LMWD frequency domain is implemented to construct two feature images by the sample of the gearbox dataset for case 2. This sample is obtained from the original signal sampled by the systematic sampling method. Compared with simple sampling, the data characteristics obtained by systematic sampling are more obvious [47]. At the same time, the sampling interval of the system sampling is 0.1 times of the period to ensure uniformity of the samples. In addition, using the same construction method, the image based on the raw data and the feature image based on Daubechies wavelet transform (DWT) are also devised to the comparative experiments in Section 4.

**Figure 7:**
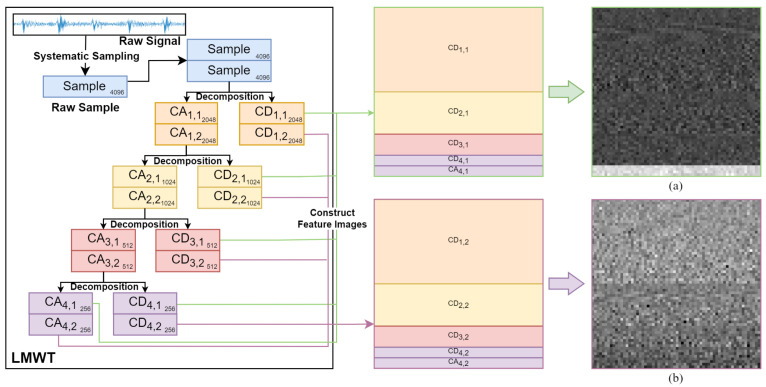
The constructed feature images from the LMWD frequency domain. (**a**) Feature image based on the first LMW base. (**b**) Feature image based on the second LMW base.

Specifically, the sample with 4096 points as shown in Figure 7 is decomposed into eight high frequency components $CD_{1,1}$, $CD_{1,2}$, $CD_{2,1}$, $CD_{2,2}$, $CD_{3,1}$, $CD_{3,2}$, $CD_{4,1}$, $CD_{4,2}$ and two low frequency components $CA_{4,1}$, $CA_{4,2}$ by two Legendre scale bases and two Legendre wavelet bases at the resolution level 4 as described in Figure 7 as follows.

More precisely, the specific steps of the constructing feature images using the LMWD frequency domain are described in detail as follows.

Step 1: The raw sample data are doubled and then decomposed by two Legendre scale bases and two Legendre wavelet bases at the resolution level 4. The length of each frequency component from the $CD_{1,1}$ to the $CA_{4,1}$ is gradually halved by the first LMW base. Similarly, the frequency component from the $CD_{1,2}$ to the $CA_{4,2}$ of the second LMW base are attained.
Step 2: For clarity, only the first LMW base is used to explain how to construct the feature image. The frequency components of different resolution levels are flattened to a feature signal with the same length as the raw sample data. Then, the feature signal is rearranged into a feature image in a matrix form with a size of 64∗64 as shown in Figure 7.

**Figure 8:**
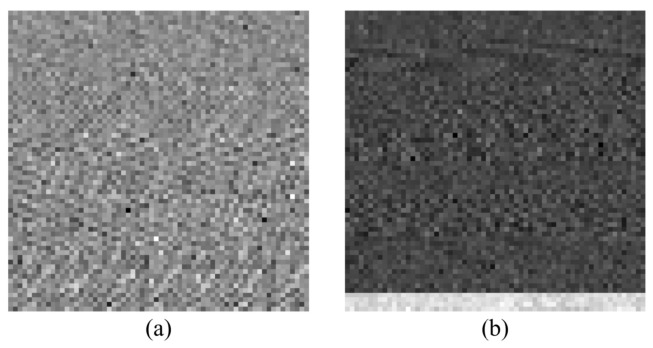
The constructed images by two methods. (**a**) Feature image based on the raw sample data. (**b**) Feature image based on DWT.

In addition, to clearly explain the feature image differences between LMW bases and the traditional methods, the method of the constructing images is also applied to the raw data and the feature signal based on DWT, and the specific results are described in Figure 8, respectively.

Finally, the two feature images obtained by LMWD are used for convolution operations in the MCNN model. Due to the various regularity and orthogonality of the LMW bases, the obtained feature images can effectively match the different fault categories of rotating machinery without loss of information. Furthermore, the feature images are easily implemented into a two-channel CNN model for effectively extracting the most valuable fault features and accurately recognizing the rotating machinery compound faults.

### 3.2. Multi-Channel CNN Based on LMWT

This subsection mainly describes how to combine LMWT with MCNN model for gearbox compound fault classification, and the following flowchart of the proposed model elaborately describes the principle of this technique.

**Figure 9:**
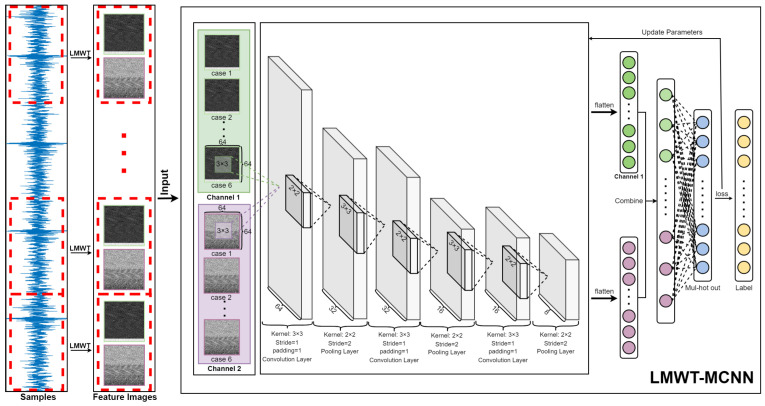
Flowchart of the LMWT-MCNN model for gearbox compound fault diagnosis.

According to the flowchart shown in Figure 9, the general steps of the proposed model are elaborately described as follows.

Step 1: The vibration signals of gearbox compound faults are sampled from the data acquisition system as shown in Figure 1.
Step 2: The vibration signals of the gearbox compound faults are divided into 600 samples according to the length of 4096 points for each sample of each fault category.
Step 3: Each sample is transformed into two feature images by LMWT using two Legendre scale bases and two Legendre wavelet bases. Then, seventy-five percent of the feature images are randomly selected for the training samples and the rest twenty-five percent for the testing samples, respectively.
Step 4: The lightweight structure of the proposed model consists of three convolutional layers with 3 ∗ 3 kernels, three batch normalization (BN) layers and three max pooling layers with 2 ∗ 2 kernels. First, the convolutional layers convolute the local regions with a series of filter kernels to generate new feature maps. The 3 ∗ 3 convolutional kernel is able to learn more excellent features with less computations relatively. Then, the BN layers are used to reduce the computational complexity of the network and accelerate network convergence. Finally, the max pooling layers perform down-sampling operations on the feature maps to decrease the size of the feature maps. The purpose of using max pooling is to extract the maximum value of the input feature map and remove the smaller noise frequency components.
Step 5: Finally, a fully-connected layer and a multi-hot cross entropy classifier are attached on the top to accomplish the compound fault recognition of the gearbox.

To summarize, each sample is decomposed into two feature images by LMWT, then the feature images are fed into the MCNN model, which addresses the lightweight combination of the LMWT and MCNN models.

### 3.3. The Flowchart of the Proposed Method Based on Edge-IOT

**Figure 10:**
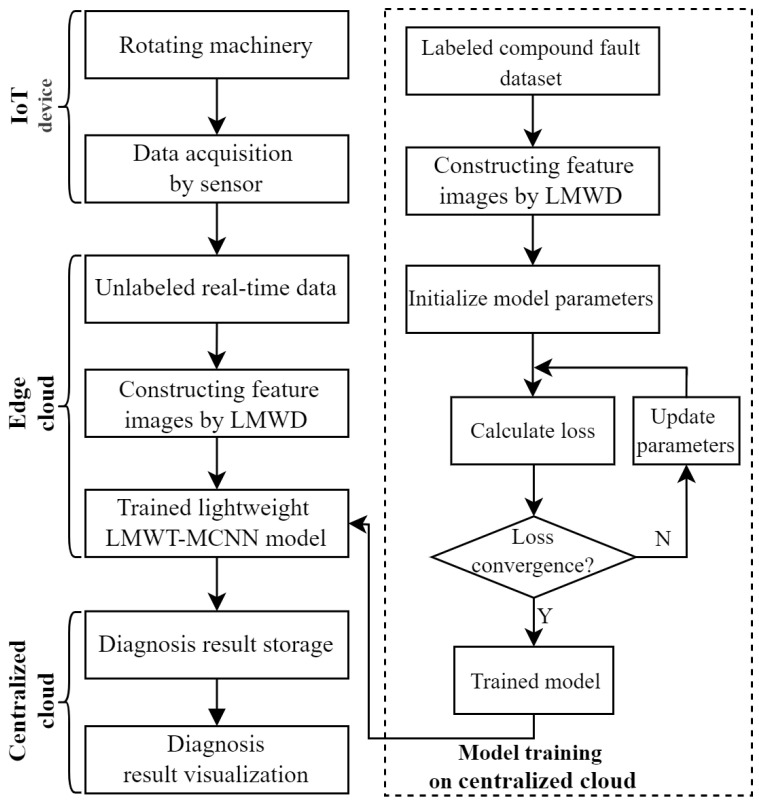
Flowchart of the proposed fault diagnosis method based on edge-IoT.

This subsection mainly discusses the overall workflow of the proposed method based on edge-IoT, encompassing the entire process from fault data acquisition to fault diagnosis and maintenance. The schematic diagram for this process is depicted in Figure 10 and is specifically divided into six steps as follows.

Step 1: The fault signals are sampled from the mechanical equipment by the sensors.
Step 2: The acquisition data are decomposed by LMWT to construct the feature images on the edge node of the edge cloud.
Step 3: The lightweight LMWT-MCNN model for fault diagnosis is trained on the centralized cloud by using labeled fault dataset. This involves initializing parameters and updating them using the loss computed from the model’s output. Once the loss of the model converges, the training process is complete.
Step 4: Then, the feature images obtained from the testing samples are fed into the trained lightweight LMWT-MCNN model downloaded from the centralized cloud to attain the highest diagnosis accuracy.
Step 5: The diagnosis results are transmitted into the centralized cloud.
Step 6: The diagnosis results and their visualization are applied to the mechanical equipment maintenance.

Finally, the two compound fault datasets of the rotating machinery are implemented to verify the effectiveness and robustness of the proposed model.

## 4. Diagnosis Results and Analysis

In this section, the PHM 2009 gearbox dataset used in this work is first described in detail. Then, the developed LMWT-MCNN model is implemented to diagnose the various compound fault categories of the gearbox. Furthermore, the diagnosis accuracy obtained by the proposed model is used to compare with the CNN method based on the raw data (Raw data-CNN), CNN method based on DWT (DWT-CNN) and other existing methods. Finally, another compound fault dataset of rotating machinery provided by Paderborn University is implemented to further verify the effectiveness of the proposed model. The comparison of the experimental results shows that the proposed model achieves the highest recognition accuracy and is more stable and robust than the existing methods. In addition, all approaches described above are implemented with Python and tested on a computer with an AMD Ryzen 7 5800H CPU @ 3.20 GHz/4.40 GB RAM.

### 4.1. Description of the PHM 2009 Gearbox Dataset

**Figure 11:**
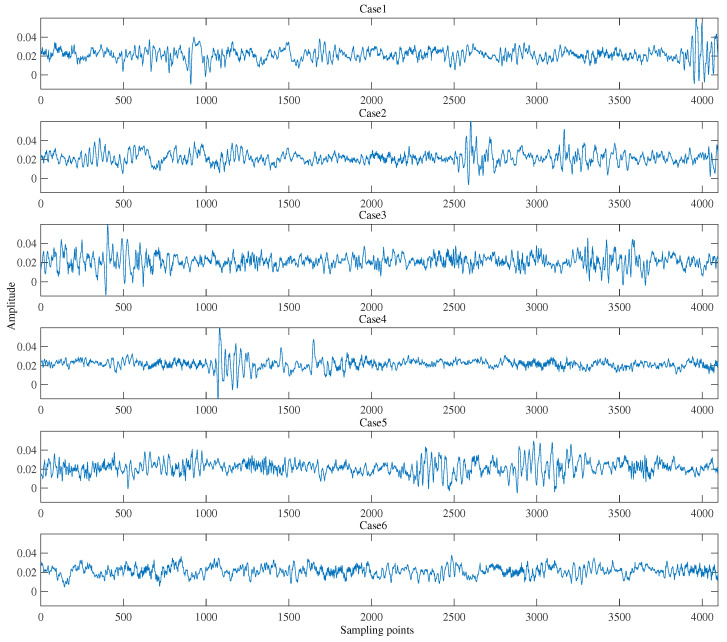
The vibration signals of the six fault types of the PHM 2009 dataset.

In this subsection, the vibration signals of the PHM 2009 gearbox dataset are described in detail, and the corresponding six compound fault categories are shown in Figure 11. Then, how to adopt the configuration parameters of the MCNN model, the training samples, and the testing samples of the proposed model for the gearbox compound fault diagnosis are specifically introduced, respectively.

**Figure 12:**
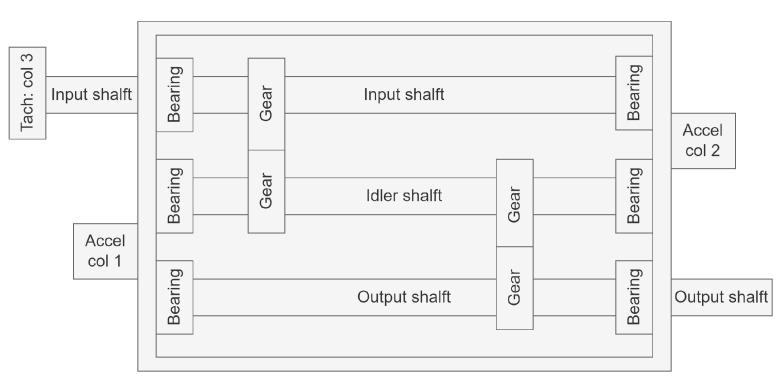
Schematic of the gearbox used in the PHM 2009 dataset.

The PHM2009 gearbox dataset is a compound fault dataset that encompasses the majority of common gearbox faults under various load conditions. The specific fault categories contain the gear chipped tooth fault, gear broken tooth fault, bearing inner ring fault, bearing ball fault, bent shaft fault, and shaft imbalance fault. Then, the fault data acquisition procedure of the PHM 2009 dataset is explained in Figure 12 as follows.

As shown in Figure 12, the structure of the data acquisition consists of the input side accelerator, the output side accelerator, and the tachometer signal. Two kinds of gears (spur gear and helical gear) are used in this data acquisition system. In this article, only the fault data from the input side accelerator and the helical gear are utilized to diagnose the six fault categories of the gearbox. For the effectiveness of classification, the six health conditions are artificially set as the corresponding multi-labels, which are described in Table 1 as follows.

The fault signals are collected at 30, 35, 40, 45, and 50 Hz speed and low load. The sampling frequency is 66.7 kHz and the sampling time is 8 s. That is, for one operating speed, there are 533,312 sample points. For each speed condition, the validation experiments in this paper use 4096 sampling points as a sample. Therefore, there exist 600 samples taken for each fault type. In order to meet the actual diagnostic requirements as much as possible, 450 samples of each health condition are randomly prepared for the training samples and the remaining 150 samples are used for testing the diagnosis accuracy of the proposed method. The specific configuration parameters of the gearbox fault data processing are elaborately demonstrated in Table 2 as follows.

Finally, the temporal waveform of the raw data samples of the six working conditions of the gearbox are described in Figure 11, respectively.

As illustrated in Figure 11, the differences between the most fault patterns cannot be easy to be distinguished. Consequently, it is very necessary to utilize the proposed model to effectively rectify different compound fault categories of the gearbox.

### 4.2. Results and Analysis

Usually, the different structures of MCNN have some impacts on the experimental results. In order to find a stable and effective LMWT-MCNN structure, the configuration parameters of MCNN should be continuously adjusted according to the diagnosis accuracy. As shown in Table 3, Model 1 has the highest diagnostic accuracy and relatively less time spent. Model 2 adds the attention mechanism (AM) after MCNN, and its diagnostic accuracy does not improve and the time cost also increases. The configuration parameters of Models 3, 4, and 5 are slightly adjusted based on Model 1, but the experimental results are not as good as those of Model 1.

**Figure 13:**
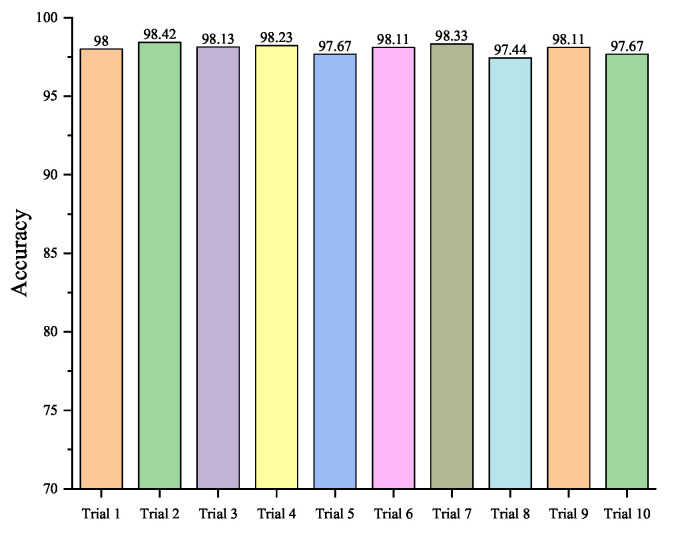
The classification accuracy of each trial by the proposed model.

Based on the comparison results, the structure of the proposed model is finally adopted as Model 1 because of achieving the highest compound fault diagnosis accuracy. In the forthcoming experiments, the optimizer for training the model will be set to Adam, with a learning rate of 0.01, a maximum training epoch of 120, and a Weight decay of 0.0005. Notably, the learning rate reduced by 30% every 30 training epochs. In addition, each experiment is repeated ten times to validate the generalizability of the proposed model, and the experiment results are shown in Figure 13 as follows.

In Figure 13, the results of ten repeated experiments conducted under the aforementioned parameter settings are presented, respectively. The average testing accuracy of the ten repeated experiments is maintained about 98.01%, and its standard deviation is only 0.32%. The standard deviation is smaller compared to other methods, which demonstrates that there are no particularity and contingency in the experiments by the proposed method.

In addition, to highlight the lightweight property of the proposed model, we compare the model with other lightweight fault diagnosis models in recent years. The comparison focuses on computational complexity and memory footprint, as displayed in Table 4. Specifically, we measure the network’s computational complexity in FLOPs and quantify the memory footprint by the number of parameters.

The results of the comparison in Table 4 significantly show that the proposed method possesses the lowest computational complexity and memory footprint. This finding indicates that our model is an exceptional lightweight model. Consequently, the proposed lightweight model can be seamlessly integrated into edge-IoT systems to achieve optimal performance in the compound fault diagnosis of rotating machinery.

Furthermore, Raw data-CNN method and the DWT-CNN method are implemented to recognize the same gearbox compound fault categories. The Precision, Recall, F-measure and Accuracy of the gearbox compound fault diagnosis results are utilized to thoroughly verify the effectiveness of the proposed method, which are described as follows.

(17) $$\begin{array}{cc} \;\mathrm{Precision}\;=\frac{TP}{TP+FP} & ,\;\mathrm{Recall}\;=\frac{TP}{TP+FN},\;\\ \;F-\mathrm{measure}\;=\frac{2\;\mathrm{Precision}\;\times \;\mathrm{Recall}\;}{\;\mathrm{Precision}\;+\;\mathrm{Recall}\;} & ,\;\mathrm{Accuracy}\;=\frac{TP+FP}{TP+TN+FP+FN}, \end{array}$$

where TP, TN, FP, and FN are the true positive, the true negative, the false positive, and the false negative, respectively. The precision measures the accuracy of positive predictions, while the recall represents the ability to identify true positive cases among correctly real positive. Then, the F-measure is a weighted harmonic average of the precision and recall, resulting in a higher value only when both the precision and recall values are high. In the end, the accuracy denotes the proportion of the correct data to the total data. Then, the testing results of different fault diagnosis methods for the gearbox compound fault diagnosis are shown in Table 5.

As shown in Table 5, the proposed model achieves the highest precision of 98.06%, recall of 98.19%, F-measure of 98.12%, and accuracy of 98.01%. In contrast, the Raw data-CNN method exhibits the lowest diagnosis accuracy of 81.33%. These comparative results demonstrate that the proposed model has the superior feature learning capability and exceptional classification accuracy.

**Figure 14:**
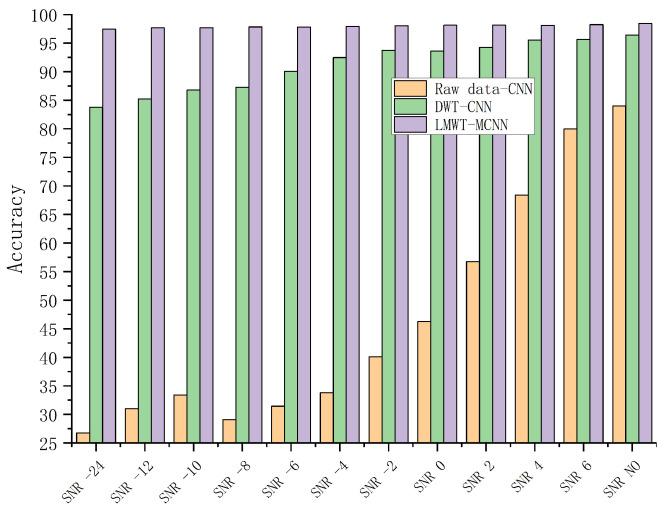
The comparison of different methods under various noisy conditions.

In the next experiment, the Gaussian white noise is added to the signals to test the anti-noise capability of the proposed model. Noise ratios ranging from −24 to 6 are applied to verify the performance of various fault diagnosis methods. The detailed comparison results by different methods under various noisy conditions are shown in Figure 14 as follows.

The diagnosis results shown in Figure 14 demonstrate that the proposed model has a strong ability to anti-noise and effectively identify each fault category of the gearbox. The proposed model achieves the best performance and more robustness in comparison with other methods under noise environment.

**Figure 15:**
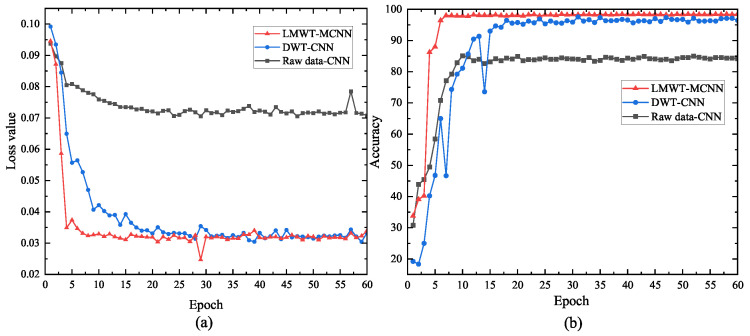
The loss and accuracy curves of different methods. (**a**) Loss curve. (**b**) Accuracy curve.

To further verify the effectiveness of the proposed method, the loss curve and iteration accuracy curve are demonstrated in Figure 15 as follows.

As demonstrated in Figure 15, the loss value of the proposed model arrives at the stable situation at about 30 iterations; the training accuracy of the proposed model attains the stable value at a few iterations, which confirm the excellent performance compared with other methods.

**Figure 16:**
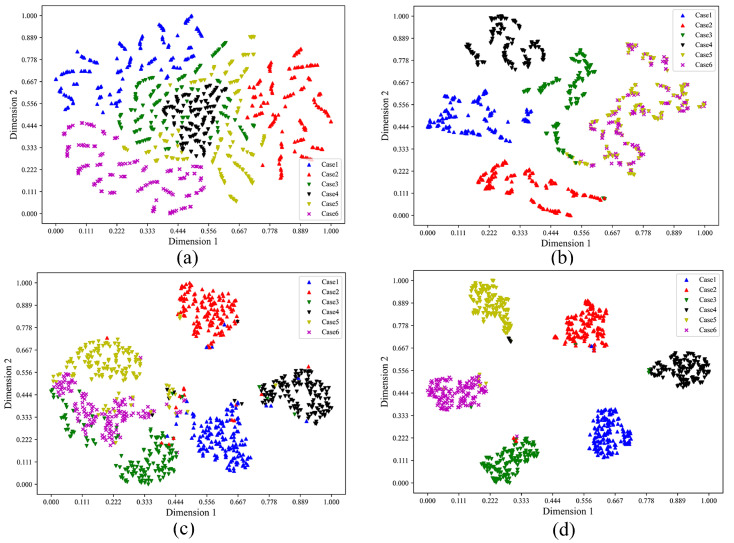
The t-SNE visualization by different methods. (**a**) Raw data. (**b**) Raw data-CNN method. (**c**) DWT-CNN method. (**d**) The proposed model.

Moreover, the t-SNE method is utilized to visualize the extracted features in two-dimensional space for different fault diagnosis methods mentioned above, which demonstrates that the proposed method has a good feature extraction ability for the gearbox compound faults. To be specific, Figure 16a shows the clustering results obtained by the raw data. Other clustering maps based on the extracted features by the Raw data-CNN method, the DWT-CNN method, and the proposed model are elaborately shown in Figure 16b–d, respectively.

From the visualized results of Figure 16a–d, it is demonstrated that the proposed model has been more effective and stable in distinguishing each fault characteristic of the gearbox than other methods. As demonstrated in Figure 16a, the data samples of the six health conditions are randomly distributed, which indicates that the difference among the raw data is small, and it is necessary to achieve effective classification by the developed methods. In Figure 16b, the features learned by the Raw data-CNN method have a better clustering than the raw data, but it is still difficult to effectively separate different fault types. In comparison with Figure 16c and Figure 16d, the two methods achieve effective separation of the six fault categories. However, in Figure 16c, there are still several samples overlapping between case 3, case 5 and case 6. As shown in Figure 16d, the proposed model almost achieves effective separation of the six compound fault types, and the distribution of the extracted features for each fault pattern is more concentrated. Therefore, the extracted features of the same health condition by using the proposed model are the best clustered.

Finally, in order to further show the superiority and effectiveness of the proposed model, other popular methods are also utilized to compare with the proposed method, and the comparison results are listed in Table 6 in detail.

The effectiveness of the proposed model in the compound fault diagnosis of the gearbox is verified in Table 6. The average accuracy of of the proposed method reaches 98.01%, which is higher than other advanced methods. Furthermore, the standard deviation of these repeated trials is 0.32%, which is also smaller than other methods as show in Table 6. The experimental results demonstrate the superiority and reliability of the proposed method.

To summarize, the above comparison results further demonstrate that the proposed model is able to effectively identify the gearbox compound health conditions.

### 4.3. Another Compound Fault Diagnosis Experiment of Rotating Machinery

**Figure 17:**
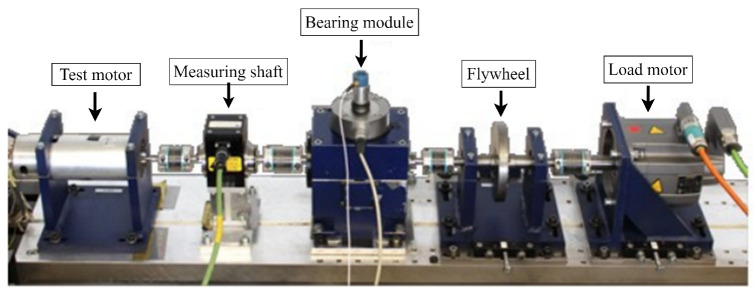
Experimental setup of Paderborn University bearing dataset.

The bearing is the important part of gearbox, thus the compound fault dataset of the bearings provided by Paderborn University is utilized to verify the generalization of the proposed model. In this dataset, all fault data are derived from accelerated life testing, including both IR (Inner Ring) and OR (Outer Ring) bearing faults. These faults include point pitting faults and plastic deformation faults on the IR and OR of the bearings. The test bench equipment consists of a permanent magnet synchronous motor, a torque measurement shaft, a test module, and a synchronous servo motor utilized as a load motor, which is described in Figure 17 as follows.

As illustrated in Figure 17, by employing a rolling element bearing module, varying test bearings are subjected to a constant radial load, thereby acquiring and storing vibration signals from the inner shell. The Paderborn University bearing dataset contains six healthy conditions and 26 damaged bearing vibration sets. A vibration transducer with a sampling frequency of 64 kHz is used to collect vibration data. The operation settings of a rotational speed of 1500 rpm, load torque of 0.7 Nm, and a radial force of 1000 N are applied in the current research.

The upcoming experiments use fault data from six different health conditions to validate the proposed model. Similar to the PHM2009 dataset, each fault type contains 600 samples, and each sample includes 4096 data points. Then, the samples are randomly divided into the training set and the testing set. The specific fault categories and samples of dataset used in this paper are shown in Table 7 in detail.

To eliminate the influence of randomness and individuality, the experiment is repeated by ten trials, and the experimental results by different methods under different noise conditions are elaborately shown in Table 8 as follows.

As described in Table 8, the proposed model achieves the highest diagnosis accuracy and the best stability compared with Raw data-CNN and DWT-CNN under different SNRs. To be specific, for the situation of −2 dB, the accuracy of the proposed model reaches 99.46%, but the accuracy of DWT-CNN method is only about 88.99%, and the lowest accuracy of Raw data-CNN is only about 75.43%. It is obvious that the proposed model also shows the best accuracy as the SNR changes from 0 dB to 6 dB than other methods. These experimental results further demonstrate that the proposed model has the excellent anti-noise ability.

Finally, to effectively show the superiority of the proposed model, other popular methods such as CNN-ELM-JDM, MPDBN-WT, AMVMD-SCNN, and ASN are also utilized to compare with each other. The comparison results of the testing accuracy are listed in Table 9 in detail.

As demonstrated in Table 9, the proposed model achieves the highest testing accuracy of 99.57% in comparison with the state-of-the-art methods. In addition, the lowest testing accuracy is 97.63% by the CNN-ELM-JDM method.

To summarize, the highest testing accuracy is achieved by the proposed lightweight model based on LMWT and MCNN compared with the existing methods. Consequently, the proposed lightweight model provides a promising technique for the implementation of online compound fault diagnosis of rotating machinery based on edge-IoT.

## 5. Conclusions

The effective and reliable intelligent fault diagnosis method based on edge-IoT is developed in this paper for the compound fault diagnosis of rotating machinery. Some comparative experiments are conducted on the PHM 2009 gearbox and the Paderborn University bearing compound fault datasets with different noise to verify the effectiveness and robustness of the proposed method. The experimental results show that the proposed method achieves the highest diagnosis accuracies of 98.01% and 99.57% without any noise, respectively, compared with the existing methods. Especially in the low signal-to-noise ratio environment, the proposed method still shows more effectiveness and robustness than other methods. To summarize, this paper proposes an effective lightweight network model for rotating machinery online fault diagnosis based on the edge-IoT context, which obtains the high accuracy, strong anti-noise ability, small storage, and low calculation costs to overcome the defects of large sensor data transmission, large cloud computing, and long-distance data transmission, and more. In future work, LMWT should be combined with other deep learning models to effectively rectify the compound fault types of rotating machinery with highly non-stationary, weak, and early faults.

## Figures and Tables

**Table 1:** Detailed description and the pattern label of the PHM 2009 dataset.

Case	Label	Description
Case 1	{1,0,0,0,0,0,0}	Label (1): Healthy
Case 2	{0,1,0,0,0,0,0}	Label (2): Chipped tooth
Case 3	{0,0,0,0,0,1,0}	Label (6): Bent Shaft
Case 4	{0,0,0,0,1,0,1}	Label (5): Ball; Label (7): Shaft Imbalance
Case 5	{0,0,1,1,0,0,0}	Label (3): Broken tooth; Label (4): Inner
Case 6	{0,0,1,1,0,1,0}	Label (3): Broken tooth; Label (4): Inner; Label (6): Bent Shaft

**Table 2:** Detailed description of the configuration parameters for the gearbox data processing.

Fault Types	Points/Sample	Samples/Fault	Training Samples	Testing Samples	Number of Wavelets	Resolution Level
6	4096	600	450	150	2	4

**Table 3:** Comparison of different LMWT-MCNN structures.

Models	Epochs	Convolution	Learning Rate	Accuracy (%)	Training Time (s)
Model 1	120	(1, 64) + (64, 32) + (32, 16)	0.01	98.33	319
Model 2	120	(1, 64) + (64, 32) + (32, 16) + AM	0.01	97.76	313
Model 3	120	(1, 32) + (32, 16)	0.01	96.78	252
Model 4	120	(1, 64) + (64, 32) + (32, 16) + (16, 8)	0.01	96.36	341
Model 5	280	(1, 32) + (32, 16) + (16, 8)	0.001	91.22	647

**Table 4:** The floating point of operations (FLOPs) and the parameter quantity (Params) of different models.

Models	FLOPs	Params
LMWT-MCNN	0.023 G	0.091 M
LMS-MAFFNet [48]	0.027 G	0.177 M
MA1DCNN [49]	0.060 G	0.850 M
ARAHNet [50]	0.072 G	7.435 M
CDCN [51]	0.053 G	0.198 M

**Table 5:** The detailed comparison results of different methods.

Methods	Precision	Recall	F-Measure	Accuracy
Raw data-CNN	78.53%	82.70%	76.66%	81.33%
DWT-CNN	95.34%	95.44%	95.28%	95.33%
LMWT-MCNN	98.06%	98.19%	98.12%	98.01%

**Table 6:** Average test accuracy and standard deviation of different methods.

Methods	Description	Accuracy (%)
Elman AdaBoost-Bagging [14]	Elman neural network + optimized AdaBoost-Bagging dual-ensemble algorithm	90.6 ± 3.62
Bi-LSTM [25]	Bidirectional Long Short-Term Memory	93.67
WT-MLCNN [33]	Wavelet Transform + Multi-label Convolutional Neural Network	94.02 ± 0.75
SA-DAL [36]	Subdomain adaptation + Deep adversarial learning	97.5 ± 5.26
TQWT+SVM [37]	Tunable Q-factor wavelet transform + Support Vector Machine	97.7
WT-RF [38]	Wavelet Transform + Random Forest	96.79 ± 1.45
LMWT-MCNN (Proposed)	Legendre MultiWavelet Transform + Multichannel Convolutional Neural Network	98.01 ± 0.32

**Table 7:** Detailed descriptions of the Paderborn University bearing compound fault dataset.

Faults	Label	Description
K001	{1,0,0,0,0}	Label (1): Normal
KA04	{0,1,0,0,0}	Label (2): fatigue: pitting in OR
KA15	{0,0,1,0,0}	Label (3): Plastic deforms: Indentations in OR
KI16	{0,0,0,1,0}	Label (4): fatigue: pitting in IR
KB24	{0,1,0,1,0}	Label (2, 4): fatigue: pitting in OR and IR
KB27	{0,0,1,0,1}	Label (3, 5): Plastic deforms: Indentations in OR and IR

**Table 8:** Average testing accuracy of the second compound fault dataset by different methods.

SNR (dB)	6	4	2	0	−2	NaN
Raw data-CNN	84.56 ± 4.68	81.04 ± 2.30	79.71 ± 3.95	77.06 ± 2.15	75.43 ± 4.04	83.72 ± 3.72
DWT-CNN	94.27 ± 3.90	92.83 ± 5.40	89.67 ± 7.25	89.06 ± 6.19	88.99 ± 7.24	96.71 ± 2.30
LMWT-MCNN	99.50 ± 0.27	99.48 ± 0.22	99.47 ± 0.15	99.47 ± 0.34	99.46 ± 0.22	99.57 ± 0.20

**Table 9:** Comparison results of several existing methods.

Methods	Description	Accuracy (%)
CNN-ELM-JDM [39]	Convolutional neural network and extreme learning machine based on joint distribution modification	97.63
MPDBN-WT [40]	Mixed pooling deep belief network + wavelet transform	98.84
AMVMD-SCNN [42]	Adaptive multivariate variational mode decomposition and multi-scale convolutional neural network	98.60
ASN [43]	Attention stream net	99.10
LMWT-MCNN (Proposed)	Legendre MultiWavelet Transform + Multichannel Convolutional Neural Network	99.57

## Data Availability

The data that support the findings of this study are available from the corresponding author upon reasonable request.
